# Resonant learning in scale-free networks

**DOI:** 10.1371/journal.pcbi.1010894

**Published:** 2023-02-21

**Authors:** Samuel Goldman, Maximino Aldana, Philippe Cluzel

**Affiliations:** 1 Department of Molecular and Cellular Biology, Harvard John A. Paulson School of Engineering and Applied Sciences, Harvard University, Cambridge, Massachusetts, United States of America; 2 Instituto de Ciencias Fisicas, Universidad Nacional Autónoma de México, Cuernavaca, Mexico; 3 Centro de Ciencias de la Complejidad, Universidad Nacional Autónoma de México, Coyoacán, Mexico City, Mexico; University of Zaragoza: Universidad de Zaragoza, SPAIN

## Abstract

Large networks of interconnected components, such as genes or machines, can coordinate complex behavioral dynamics. One outstanding question has been to identify the design principles that allow such networks to learn new behaviors. Here, we use Boolean networks as prototypes to demonstrate how periodic activation of network hubs provides a network-level advantage in evolutionary learning. Surprisingly, we find that a network can simultaneously learn distinct target functions upon distinct hub oscillations. We term this emergent property *resonant learning*, as the new selected dynamical behaviors depend on the choice of the period of the hub oscillations. Furthermore, this procedure accelerates the learning of new behaviors by an order of magnitude faster than without oscillations. While it is well-established that modular network architecture can be selected through evolutionary learning to produce different network behaviors, forced hub oscillations emerge as an alternative evolutionary learning strategy for which network modularity is not necessarily required.

## Author summary

Large networks of interconnected components such as genes or even machines can coordinate complex behavioral dynamics. One outstanding question has been to identify the design principles that allow networks to learn new behaviors. Here, in simulating learning cycles, we randomly modify the interactions between the network components and select networks that exhibit a desired behavior. Surprisingly, we find that new behaviors can be learned faster when the state of the most connected network node (hub) is forced to oscillate during learning than when it is not oscillating. Hence, we call this new property *Resonant learning*. Remarkably, using distinct periods of forced oscillations allows a given network to learn distinct behaviors. Resonant learning may be relevant to the evolution and adaptation of a range of biological systems that require periodic stimuli to reconfigure their dynamics in order to cope with new environmental challenges.

## Introduction

Over the last two decades, several experiments have revealed that many large biological networks follow a scale-free topology characterized by the existence of a few highly connected network nodes (hereafter called *hubs*) that govern most of the network activity [1,2]. For example, cellular transcriptional networks have evolved such that a small number of “master regulator” genes can control the expression of a large number of downstream genes [3,4]. Recent experimental advances have enabled several groups to monitor long time series of expression (activation) of these master regulators [5] and to report the existence of pulsatile behaviors with varying timescales [6,7]. Overall, these studies suggest that specific timescales of pulses are likely associated with corresponding dynamical behaviors of the cell [7–12].

A plausible hypothesis is that the multiple timescales associated with pulses of master regulators’ activity may have been selected by evolution to allow cells to adapt to a range of fluctuating environments such as feeding patterns, circadian rhythms, or even ecological times. The selectivity of distinct cellular behaviors by the means of distinct timescales would have emerged then as a key property of crucial importance for the survival of organisms, as discussed in a series of recent works [13–16]. While some benefits of “master regulatory” and scale-free network topologies are now well known, the interplay between the scale-free network topology, master regulators with pulsating activity, and the evolution of complex behavior remains poorly understood at the systems level. Oscillatory behavior is important not only for gene regulation networks, but more generally, for signal transduction pathways which control important cellular processes such as growth and differentiation [17].

While it is often difficult to predict the behavior of such large intricate networks, it is standard practice to use Boolean network models to simulate the complexity of interactions between complex network elements [18]. Despite their simple rules, Boolean networks have emerged as powerful systems to successfully approximate the dynamical behavior of many technological and biological systems with complex time-dependent behaviors as diverse as social interactions [19], gene regulations [20–22], electric grids [23] and industrial processes [24]. Beyond simulating network function, such Boolean networks can also be perturbed and evolved toward target behavior to study and understand real-world evolutionary and adaptation processes with control parameters that often remain unknown [25–27].

Inspired by these biological considerations, we ask whether Boolean networks with scale-free topology could be evolved and learn new complex behavior such that specific external timescales can be associated with certain dynamical behaviors. Operationally, we model the effect of external timescales by applying an oscillatory periodic signal with a certain timescale onto highly connected nodes of the network, such as the master regulators. We ask how this procedure can contribute to coordinate desired behaviors of such large non-linear dynamical systems. To be more explicit, let us assume that some new environmental challenge requires the network to develop an internal response, which we call the target response function *F*(*t*). At the same time, the network receives, throughout one of its hubs, a periodic external input signal *I*(*t*). We want to answer the following question: What characteristics should the input signal *I*(*t*) have to make the cell evolve in the most effective way towards the target behavior *F*(*t*)?

Understanding how networks adapt or can evolve over time in response to periodic perturbations has been a rich area of research. Both noise and small perturbations applied periodically to network nodes can drastically change the outcome of networks and their evolution. Stern implemented an evolutionary algorithm using populations of Kauffman networks to demonstrate that “noisy signals” applied to randomly selected nodes, repeated over several generations of evolutionary learning rounds, produce desired behavior which is not recovered when the noisy input signal is removed [28]. From a control perspective, Cornelius et al. demonstrate a strategy to use perturbations to guide a network toward a desired state when only a small set of accessible nodes in the network can be modified, parallel to real world settings such as therapeutic design, when only a subset of genes or gene products can be drugged effectively to “save” the network [29]. Closer to our approach, a separate line of investigations has revealed how oscillatory inputs, often referred to as “frequency-modulated” signals, can be interpreted in small circuit settings. Gao et al. consider how small subnetwork motifs can process and respond to complex input signals, and Rue et al. observe that individual nodes in Boolean networks can relay the frequency of an incoming temporal signal [30,31]. However, to our knowledge, none of these studies specifically attempted to characterize how oscillatory input signals could be used in network evolutionary learning. It is not clear either how sustained oscillatory input signals on hub nodes like the ones observed in experiments can shape the dynamics of large dynamical systems such as random Boolean networks. Here, using an evolutionary algorithm, we find that these networks can learn distinct target behaviors when hubs are forced to pulse with distinct periods, whereas when there is no oscillation, they cannot. We term this new property of network-based learning in the presence of input oscillations, *resonant learning*.

## Network model

We use random Boolean threshold networks (RBTN) as prototypes for the study of large dynamical systems that include feedback loops [32,33]. RBTN’s have successfully been used to reproduce experimentally observed gene expression patterns in some organisms [34,35]. We define networks with *N* nodes, {*σ*_1_, *σ*_2_,…,*σ*_*N*_} and a set of directed edges, *E*, $\left\{(\sigma_{i_{1}},\sigma_{j_{1}}),(\sigma_{i_{2}},\sigma_{j_{2}}),\ldots ({\sigma_{i}}_{\left|E\right)},\sigma_{j_{\left|E\right)}})\right\}\in E$. At any timestep, each node *σ*_*i*_ can acquire the values 0 or 1, corresponding to the expression states *OFF* (inactive) and *ON* (active), respectively. The state of the entire network at time *t*, which we refer to as ***σ***(***t***), is the state at time *t* of all the genes: ***σ***(***t***) = {*σ*_1_(*t*), *σ*_2_(*t*),⋯,*σ*_*N*_(*t*)}.

**Fig 1:**
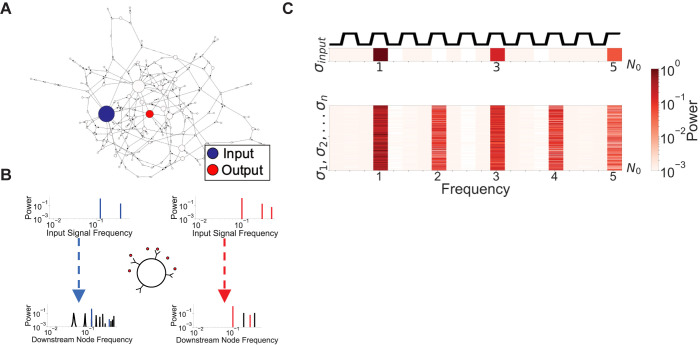
**Effect of hub oscillations on downstream nodes (A) Illustrative example** of network topology in which downstream nodes (red) respond to a given oscillatory input hub behavior (blue). (**B) Single Node Frequency Response.** Power spectrum (bottom) calculated from a randomly selected downstream node’s time series when hub node oscillates at specific frequencies (top). The plotted frequency here is directly related to the period, ($T=\frac{1}{f}$). The power spectra of output nodes exhibit both higher and lower frequencies than the input signal. The corresponding time domain sequences can be found in the SI (**Fig 3 in S1 Text).** (**C) Input-output relationship network response.** The frequency response of this same network is shown for all non-frozen nodes (rows) when the input hub oscillates with a frequency of *f = 0*.*1*, equivalent to *T = 10*. The input square wave time series and its associated power spectrum are shown above the output power spectrum for clarity. The frequency axis is normalized with the input frequency of the hub, *N*_0_ = *f* = 0.1, showing that harmonics dominate the behavior of downstream nodes. This procedure is repeated for input period *T = 8* in the SI (**S4 Fig**).

The state of each node is determined by its set of regulators (or *inputs*). However, to construct networks with topologies statistically analogous to the ones observed in real organisms, instead of assigning a set of inputs to each node *σ*_*i*_, we better assign its outputs (the set of nodes that *σ*_*i*_ regulates). By assigning the outputs to each node in the network, the inputs of all nodes are then determined (and vice versa). We choose scale-free network topologies where the number of out-degree connections per node is randomly distributed according to the power-law distribution *P*(*k*) = *Ck*^−*γ*^, where *C* is a normalization constant and *γ* is the scale-free exponent that characterizes the network topology. If the *k*_*i*_ outputs of node *σ*_*i*_ are chosen randomly from anywhere in the network, only the out-degree topology is scale-free, whereas the in-degree topology is homogeneously random (Erdös-Rényi) with Poisson distribution. This topology, which is shared by a large number of biological networks [36], confers the desirable property of “hubs” regulating a large number of down-stream nodes [37–39] (**Fig 1A**). For example, in regulatory transcriptional networks, these hubs could represent central endogenous regulators such as sigma factors. Moreover, we previously showed that scale-free networks evolve more efficiently toward a target function than homogeneous networks, which makes scale-free networks potentially good candidates to identify evolutionary learning properties of large dynamical systems [40,41].

The updating rule for the state ***σ***(***t***) a RBTN is based on a simple threshold activation function (**Fig A in S1 Text**). We define the updating rule such that the state of any node at the next time *t*+1 step is determined by the product between the weights of incoming connections and the state of the input nodes at time *t*:

$$\sigma_{i}(t+1)=\left\{ \begin{array}{c} 0,\;if\;\sum_{j=1}^{N}w_{ij}\sigma_{j}(t)<0\\ 1,\;if\;\sum_{j=1}^{N}w_{ij}\sigma_{j}(t)>0\\ \sigma_{i}(t),\;if\;\sum_{j=1}^{N}w_{ij}\sigma_{j}(t)=0 \end{array}\right.$$ (1)

The weight *w*_*ij*_ for each directed edge in the network from node *σ*_*j*_ to node *σ*_*i*_ is drawn from a distribution *P*(*w*) uniform on the interval [−1,1], whereas *w*_*ij*_ = 0 if there is no connection between *σ*_*j*_ and *σ*_*i*_. Because the signal that each *σ*_*i*_ receives from its regulators is additive, each weight *w*_*ij*_ exerts either an inhibitory or activating effect on its target node with varying strength. Negative weights correspond to inhibitory interactions whereas positive weights correspond to activating interactions. It is known that some repressors, when inactive, have a positive effect on the target gene. The updating rule takes this effect into account. To see this, let us assume that *σ*_*j*_ is a repressor of *σ*_*i*_. The corresponding weight *w*_*ij*_ is negative. When the repressor *σ*_*j*_ is absent (*σ*_*j*_ = 0), the weight *w*_*ij*_ does not appear in the sum in Eq (1), which effectively contributes to a more positive value of the sum and makes it more likely for the regulated gene *σ*_*i*_ to be in the active state. An analogous situation occurs when an activating regulator (with a corresponding positive weight) is absent. Therefore, the updating rule in Eq (1) mimics a biological regulatory network in which genes are only active if the combined effect of its regulators, whether active or inactive, is positive. Given the discontinuity apparent in Eq (1), the dynamical behavior of the network is highly nonlinear. Altogether, this simple non-linear rule can generate a rich variety of dynamical behaviors and reproduce experimentally observed gene expression patterns [34,35,42].

Note that the dynamic updating rule given in Eq (1) is deterministic. And since the total number of possible network states is finite and equal to 2^*N*^, starting from one initial condition ***σ***(**0**) = {*σ*_1_(0), *σ*_2_(0),⋯,*σ*_*N*_(0)} after some iterations the network will inevitably fall into a previously visited state. From that point on, the network will travel through a set of network states that will repeat forever. This set of states is called an *attractor*. One network can have several attractors. Which attractor the network falls into depends on specific initial conditions. Different initial conditions can lead to the same attractor or to a different attractor. The set of all the possible attractors is known as the *attractor* landscape (in analogy with the epigenetic landscape introduced by Waddington). It was first hypothesized by Kauffman and then experimentally shown by Huang et al. that the dynamical attractors of the network may correspond to different cell types or cell fates [43]. Therefore, controlling the behavior of the dynamical attractors of the network with external perturbations is of great importance.

## Methods & results

### Hub oscillations propagate in downstream nodes

First, we should be reminded that Boolean Threshold Networks exhibit a second-order phase transition from ordered to chaotic dynamics (see [32] and Fig B **in S1 Text**). This phase transition is controlled by two parameters: the average input connectivity *K*_*I*_ (the average number of regulators per node), and the asymmetry parameter *α*_*w*_ = *μ*_*w*_/*σ*_*w*_, where *μ*_*w*_ is the average of the weight distribution *P*(*w*) and *σ*_*w*_ its standard deviation. The average input connectivity *K*_*I*_ is in turn determined by the scale-free exponent *γ*. In what follows we will analyze how the attractor landscape changes when the main hub of the network is perturbed with an oscillatory signal. All the results presented in this work correspond to *α*_*w*_ = 0 to consider similar fractions of activating and inhibitory interactions.

To probe the effects of hub oscillations on the network nodes and attractor dynamics, we simulate large scale-free networks (*γ* = 1.9) in which the hub node *σ*_*hub*_, the node with highest outgoing connectivity, is forced to oscillate periodically at a pre-set switching rate. We present results when only the main hub is forced to oscillate to analyze the simplest but important case of the evolutionary learning process with the minimum of external perturbations. Clearly, the more hubs are forced to oscillate, the more impact they will have on the dynamics. We choose the scale-free exponent *γ*≈1.9 because it corresponds to the critical-like regime of a second order phase transition as shown in **Fig B in S1 Text,** where learning algorithms are the most impactful (**Fig G in S1 Text**) [41]. Within networks, a hub, *σ*_*hub*_, is selected as an input alongside an arbitrarily chosen response node *σ*_*r*_, the “reporter” or output node. We use periodic and symmetric square waves with period *T* as the driving input signal of the hub (**Fig 1A**). Surprisingly, we find that distinct time series outputs (attractor cycles) associated with the reporter node (**Fig 1A,** red) in forced networks can be induced by distinct input periods of oscillation applied to *σ*_*hub*_. Given that *T* only defines the rate at which *σ*_*hub*_ switches between *0* and *1* states, it is surprising that such hub forced oscillations can induce drastically different network cycles.

We first confirm that such behaviors are not a consequence of redefining the position of the phase transition threshold due to the external perturbation. To this end, we simulate the ordered-to-chaotic phase transition by computing the hamming distance between two different network trajectories starting from two slightly different initial conditions. (A network trajectory is the series of states ***σ***(**0**)→***σ***(**1**)→***σ***(**2**)→⋯***σ***(***t***)→⋯***σ***(∞) that are obtained from repeated iterations of the dynamic updating rule given in Eq (1), starting from the initial condition ***σ***(**0**).) We find that this transition occurs at *γ* = 1.9 both in the presence and absence of hub node oscillations (**Fig B in S1 Text**).

To visualize that the network has reached a stable, new attractor cycle upon sustained hub oscillations, we monitor the response of single nodes of the network to various input periods *T*. That is, we allow a network to update over many time steps, under the restriction that *σ*_*hub*_ oscillates at a specific period *T*. Exciting *σ*_*hub*_ at a certain period *T* would yield new time series for each node *σ*_*i*_ in the network. To evaluate the degree to which downstream nodes either transmit the same or different frequencies as those driving the oscillatory hub, we compute the power spectral density (**Fig D in S1 Text**) of the time series associated with each node *σ*_*i*_. By inspecting the case of a single downstream node, we can see that when the input signal has a period of *T* = 8 (Blue), the network produces harmonics and subharmonic response frequencies (Fig 1B). On the other hand, when the input period is *T* = 10 (red), the downstream node only exhibits harmonics. The important point to notice is not the exact value of the period *T*, but the fact that different periods of input oscillations on the hub node can induce a range of downstream behaviors on the output nodes. For the sake of clarity, we first show a specific example (**Fig 1B**). For clarity, we define resonant frequencies in the network as multiples of the input oscillation frequency, $N_{0}=f_{input}=\frac{1}{T}$. We can convert any resonant frequency back to the time domain by inverting it, giving resonant periods. For period *T = 10*, the input frequency is *N*_0_ = 0.1, and a resonant frequency is 5*N*_0_ = 0.5, which corresponds to a resonant period *T*_*res*_ = 2.We then summarize the general behavior of the downstream nodes using a heatmap that depicts the power spectra for all non-frozen nodes that switch back and forth between ‘on’ and ‘off’ states (**Fig 1C)**. Depending on the value of the scale-free exponent *γ*, a fraction of the network nodes will remain “frozen” throughout the dynamic trajectory. In the ordered regime, almost all the nodes will remain frozen either in the value 0 or in the value 1. In the chaotic regime, almost all nodes will change their values throughout time. At the critical point *γ*≈1.9 a fraction of the nodes will remain frozen, and the complementary fraction will change in time. This fraction fluctuates widely depending across network realizations and initial conditions. We observe that while the oscillations of the controlling hub have a fixed single timescale, downstream nodes oscillate with a range of frequencies that differ from that of the forced oscillating hub.

### Hub oscillations induce resonant attractor cycles

After observing that individual nodes respond distinctly to a given input period, we ask whether we can observe more general behaviors when we consider the state of the system at the level of the state ***σ***(***t***) of the whole network. As it was mentioned in the Model Network section, the updating rule for our networks (Eq (1)) is deterministic. Therefore, the state space has a finite number of states and consequently the network will eventually enter a fixed cycle of states, which is known as an attractor cycle. We define the start *t*_1_ and end *t*_2_ of the attractor cycle to be the first two time points at which the states ***σ***(*t*_1_) and ***σ***(*t*_2_) become equal: ***σ***(*t*_1_) = ***σ***(*t*_2_), and all network states prior to time *t*_1_ belong to the transient states of the network (see also ***Defining an Attractor* in S1 Text** for further specification). We use attractor cycles as means to characterize the overall dynamical behavior of the network. We then ask how attractors associated with a given network are perturbed in response to oscillating inputs.

We first specify a null experiment for comparison. Namely, because the oscillating input signals involve switching between the *0* and *1* state of the hub node, we define ground-state attractors as the set of all attractor cycles when the hub node of the network is blocked, made to stay constant, either in the *0* or *1* state. That is, we can sample many different initial conditions for a given network and force *σ*_*hub*_(*t*) = *const*. The network states and the progression they follow over time define the ground-state basins of attraction for a given network. We define the ground-state attractors of the network as the ones that are reached when the hub is frozen either at 0 or at 1 by analogy with the fact that in unperturbed cells, several important genes are constitutively expressed or are constantly unexpressed. For these genes to change their expression behavior, the cell must be perturbed with external stimuli. An analogous situation occurs with signaling pathways, which are often triggered by constant external stimuli.

**Fig 2:**
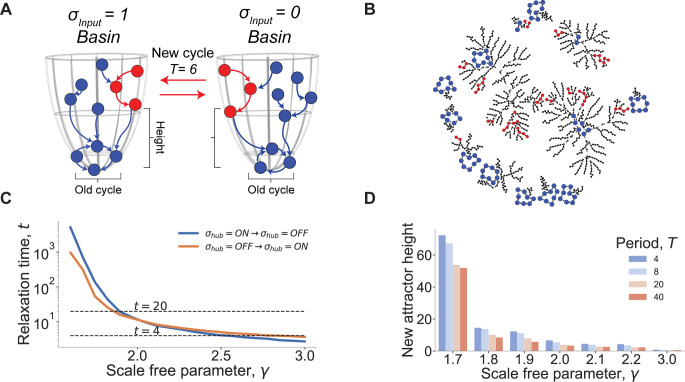
Creating new attractor landscapes. **(A) Oscillating hub creates new attractor cycles.** In a non-oscillating setting, network states (blue) will converge on deterministic attractor cycles (bottom of funnel). However, by oscillating *σ*_*hub*_ new attractor cycles (red) are created. New cycles are separated from the ground state cycle by a non-zero height that represents the number of states for the new attractor to relax to the ground state when the hub stops oscillating. **(B) Visualizing a real attractor landscape.** A sample network with *N* = 1000 and *γ* = 1.9, is probed from various input conditions when the network state is fixed to either the ON or OFF state. Arrows represent network state transitions and attractor cycle states are shown in blue for this network when the input is blocked at either an ON or OFF state. New attractor states that appear when the input node oscillates are also shown in red, with an example attractor cycle for input oscillation *T* = 4 highlighted with a cycle. (**C) Relaxation time of the network.** The average relaxation time, *t*, is depicted against the scale-free parameter *γ* for networks of 1000 nodes. The relaxation time defines the time it takes for the network to settle onto a new ground state cycle when the hub is switched from *σ*_*hub*_ = 1 to 0 (and vice versa). (**D) Heights of new attractors as a function of the scale-free parameter and the period of hub oscillations.** For each *T* = 4, *T* = 8, *T* = 20, and *T* = 40, the average height of the new attractors from 10 distinct networks is plotted, (1,000 random initial conditions). New attractor cycles for more chaotic networks (*γ*>1.9) yields larger heights i.e. new cycle states are further out in the reference basins.

Each network state (initial conditions) will eventually converge upon an attractor cycle according to this null reference scheme, and we can quantify how far away each network state is from its corresponding attractor as the number of time steps it takes to converge to that attractor (**Fig 2A)**. We call this distance the “height” of any given network state. In this scheme, the network states that are part of attractor cycles are considered to have a height of *0* when the hub is blocked to a fixed value. We stress the fact that attractors represent cell types or cell fates. Therefore, a network that is already cycling in one of its attractors represents a specific functional state of the cell. The effect of oscillating the controlling hub becomes analogous to exciting the network that bounces back and forth between these ground-state landscapes defined when the hub state is fixed (either ON or OFF). Interestingly, we find that this procedure yields new attractor cycles that share some states with the reference basins of attraction but also that show new ones (**Fig 2A**). As a result, when the hub node is forced to oscillate, the network converges upon new attractor cycles that have non-zero average height. This effect can be seen in practice by creating an attractor landscape visualization for a sample network (N = 1000, *γ* = 1.9). When the input node is forced to oscillate, new attractor cycles are created from network states that were not previously participating in cycles (red nodes) (Fig 2B).

Next, we characterize the longest oscillation period that can yield new attractor cycles. We reasoned that if the period of switching *σ*_*hub*_ between the *0* and *1* states is too long, the network will exhibit the limit behavior with the same attractors as if *σ*_*hub*_ were either blocked at *0* or *1*; under this condition no new attractor cycle behavior will be reached. To identify the longest possible oscillation period that can yield new attractors, we simulate a random initial condition, block the hub in the *0 (*or *1)* states, and allow the network to fall onto an attractor cycle. Then, we switch the input hub node to the *1* (or *0)* state and measure the relaxation time for the network to reach a new attractor cycle with the new hub state. We repeat this procedure over *1*,*000* different networks and initial conditions to obtain the average relaxation time for various *γ* exponents. We consider this relaxation time to be an estimate for evaluating the longest period duration beyond which the network will be insensitive to the oscillations of the hub (**Fig 2C**). Networks with *γ* = 3.0 have relaxation time, *t*_*relax*_ = 4, therefore the longest period for the hub oscillations capable of inducing new behavior is twice this relaxation time *T* = 2*t*_*relax*_ = 8. Additionally, we find the longest relaxation time for networks that have a regime close to critical-like behavior (*γ* = 1.9) to be *t*_*relax*_ = 20. Therefore, any period greater than *T* = 2*t*_*relax*_ = 40 will yield no new attractor cycles and will be associated with a *0* height regardless of how long the oscillating period is.

Using the average “height” as a metric, we construct a phase-diagram to illustrate how statistically different the new attractor cycles are from the initial ground-state cycles as a function of the scale-free exponent, *γ*. To this end, we generate and examine networks for different values of *γ* in the interval [1.7,3], in the presence of an oscillating *σ*_*hub*_ with different input periods. As expected, networks with larger *γ* are more ordered and fall on ground-state attractor cycles with height close to *0*, whereas more critical and chaotic networks with *γ*≤1.9 exhibit novel attractor cycles composed of network states with greater heights (**Fig 2D**). This diagram demonstrates that critical dynamics become necessary to innovate attractors when longer oscillatory inputs are considered. By contrast, sparsely connected networks yield trivial behavior even in the presence of rapid oscillating inputs (shorter timescale). Altogether, these observations point to the explanation that biological networks may have converged upon critical regimes with topology *γ*~1.9 because they are dynamically more malleable and respond to external temporal inputs with a wider range of behaviors without being extremely sensitive to changes in the initial condition. Overall, newly generated attractors are in fact novel with respect to each other and with respect to the ground-state attractors as long as the network is sufficiently critical with *γ*<1.9 (**Figs E-F in S1 Text**), which also ensures that different input hub periods can induce different attractor cycles (**Fig 2B)**.

Not only are these attractors selectable by specifying a certain input period, but we find that these newly selected attractors are robust to variations in initial conditions. Specifically, we can estimate this robustness as the number of new attractor cycles identified by sampling a large number of initial conditions. We draw random initial conditions for each network and input period, and we determine the fraction of unique attractors. With *γ* = 1.9 in the critical regime, we find that on average fewer than 20% of initial conditions yield distinct attractors, indicating the robustness of this landscape (**Fig G in S1 Text**). In Boolean scale-free networks, new attractor cycles can be induced by simply changing the duration of square wave periods for a single input hub node. These new attractor cycles are robust to different initial conditions and are mainly governed by the duration of the period of hub oscillations. We call *resonant* attractor cycles these new network behaviors induced by forcing periodic inputs on the hub. This seemingly simple conclusion shed light on a new emergent dynamical behavior: oscillations of a central regulatory hub can induce entirely different network behaviors represented by new and distinct attractors.

### Resonant attractor cycles exhibit fast learning dynamics

So far, we have just analyzed the effect on the network dynamics of forcing the hub node to stay constant or to oscillate with a given frequency. In what follows we perform classic evolutionary experiments to train (adapt) networks to learn a predefined behavior in the presence or absence of oscillatory external signals. For this, we implement populations of networks which evolve independently from one another. The main hub of each network is forced to oscillate at a given fixed frequency. Each network in the population is mutated and then we check whether the reporter node exhibits a behavior that is closer or not to the predefined target behavior. Networks whose behavior gets closer to the target behavior are reproduced and conserved to pass to the next generation, while networks that deviate from the desired behavior are discarded. In this way all the networks in the population will eventually converge and learn the desired behavior.

It has already been well-established that scale-free networks can evolutionary learn new behaviors faster than homogeneous networks [41]. However, we now ask whether new resonant attractor cycles can also learn new target functions. We thereafter follow an evolutionary learning scheme [41]. We first initialize a population of *N*_*pop*_ = 50 scale-free networks near the critical regime with *γ* = 1.9. Given the expense of large network simulations, we use networks of size *N* = 500, which we confirm have near equivalent evolutionary performance to larger networks (**Fig H in S1 Text**). We validate that networks with this parametrization are both adaptable (i.e., evolve quicker than more ordered networks) and have attractor cycles with relatively few states (**Figs I and J in S1 Text)**.

The goal of this learning algorithm is to evolve the network behavior in such a way that a single output node of the network exhibits the same time series of “on” and “off” states as that of the target function, which is a predefined fixed random sequence of 0’s and 1’s of length *L*_*c*_ (**Fig K in S1 Text**). Such a target function represents a complex network response, which schematically models what a biological system would need to accomplish upon changing environments. Both the output node, which is selected randomly, and the target function remain the same throughout the learning cycles. We perform each simulation for *G* = 10^5^ generations. In each learning generation, we generate *M* = 3 mutated networks from each of the *N*_*pop*_ networks. We score how well all networks, including the unmutated networks, have learned all the target functions. We select the top scoring *N*_*pop*_ networks from the temporary population of size 4*N*_*pop*_ (parent and mutant networks, **Fig L in S1 Text**), and repeat this process for all the *G* = 10^5^ generations.

To mutate each network, we use a fixed mutation rate of *μ* = 0.02, such that with probability *μ* we mutate each node in the network, using the same optimized conditions as in [41] with equal probability. A mutation for some node involves changing the weight or target node of an outgoing edge. By only modifying the target node or weight, we guarantee that we maintain the power-law out-degree distribution of the network.

**Fig 3:**
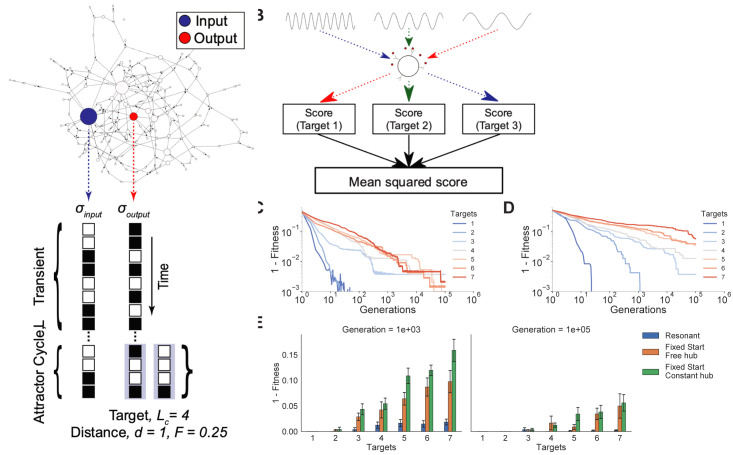
Network evolution in the presence of oscillating hub. **(A) Fitness function for learning multiple targets.** An arbitrarily selected output node downstream in the network is scored by its similarity to a randomly generated target function (see SI for more details**). (B) Learning multiple functions.** The network learns multiple random target functions to match corresponding input periods. The hub node oscillates successively at all pre-selected input periods, *T*_1_, *T*_2_,..,*T*_*M*_ in separate runs beginning from random conditions. In each learning cycle, we score how close the attractor cycle at the output node matches the corresponding target function *f*_1_(*t*), *f*_2_(*t*),..*f*_*M*_(*t*) by its mean squared fitness function across all trials. **(C) Scale-free networks with oscillating hub can learn multiple target functions.** 10 different simulations with *N* = 500, *γ* = 1.9, show that the network can learn a maximum of 7 different target functions associated with 7 input frequencies before a loss in performance. To decrease variance emerging from trial-to-trial variability, behavior is averaged over 30 trials for 1–3 targets, 20 trials for 4–5 targets, and 10 trials for 6–7 targets. **(D) Networks with a hub that has a fixed state learn much slower.** Rather than allowing the network to learn in the presence of an oscillating hub with pre-selected period, a single fixed state for the hub and one target cycle are provided, like the previous learning procedure (forcing the hub node to stay constant). Interestingly, while we might expect the network to learn faster when it must only be concerned with as few as 3 of the 2^500^ total input states, the network learns far slower under this alternative scheme, than in the presence of an oscillatory hub. **(E) Summary plots of results from (C) and (D) for the learning cycle 10**^**3**^ **and 10**^**5**^. Networks with the resonant learning scheme are more fit than networks that must learn with a non-oscillating hub, regardless of whether the hub node is fixed or allowed to update freely. Bars are plotted with standard error.

To score the performance of a given network in a generation, we randomly choose an initial condition for the network (i.e., a set of “on” and “off” states for each node). Then, oscillating the input node with input period *T*, we allow the network to update through time until it falls onto an attractor of length *L*. We take the time series of the output node in this attractor cycle and estimate the distance between this time series versus the target time series of length *L*_*c*_ (**Fig 3A**). To be more specific, let $f=\{\hat{\sigma }(t_{1}),\hat{\sigma }(t_{2}),\cdots ,\hat{\sigma }(t_{L_{c}})\}$ be the target timeseries we want the reporter node to follow during *L*_*c*_ timesteps (each value $\hat{\sigma }(t_{i})$ has been previously defined to be 0 or 1), while $\sigma_{out}=\{\sigma_{out}(t_{1}),\sigma_{out}(t_{2}),\cdots ,\sigma_{out}(t_{L_{c}})\}$ is the actual value of the reporter node under the network dynamics during these *L*_*c*_ timesteps. To determine how well the reporter node is reproducing the desired signal, we compute the Hamming distance between these two timeseries as follows:

$$h=\frac{1}{L_{c}}\sum_{i=1}^{L_{c}}\left|\hat{\sigma }(t_{i})-\sigma_{out}(t_{i})\right|.$$

Because we want this scoring function to be invariant to the offset of the attractor cycle time series, we consider all circular permutations over the target function (starting from a random initial condition, a periodic attractor with period *L* can be reached at any of its *L* constituent points). Additionally, it is often the case that *L*≠*L*_*c*_, so we repeat both time series to a length *L*′ = *min*(*L*, *L*_*c*_). By doing this, we avoid penalizing the attractor cycle for a length mismatch with the target function. Therefore, to avoid this mismatch, in the previous equation we compute the Hamming distance *h* using *L*′ instead of *L*_*c*_. Thus, we define our score for a single target function of length *L*_*c*_, the *Fitness* of the evolutionary process, as the complement *Fitness* = 1−*h* of the averaged hamming distance *h* between the target function and the output node’s time series (**Fig K in S1 Text**). Thus, if the Hamming distance has its minimum value *h* = 0 (the two signals are identical), the fitness reaches its maximum value *Fitness* = 1, whereas when the Hamming distance acquires its maximum value *h* = 1 (maximum discordance between the output behavior and the desired one), the fitness is *Fitness* = 0. Note that we have three important parameters here: The period *T* of the external signal forcing the hub node, the length *L*_*c*_ of the desired target function, and the period *L* of the attractor under the periodic forcing signal.

At each generation, only the *N*_*pop*_ networks (out of 4*N*_*pop*_) with the highest values of *Fitness* will pass to the next generation **(Fig L in S1 Text)**. Furthermore, target functions that are too long are also difficult to learn, especially when their lengths exceed that of the relaxation time of the network (i.e. the average “height” of the attractor landscape, see **Figs 2B and M in S1 Text)**. Therefore, we restrict our learning algorithms to input periods *T < 20* for networks with *N* = 500. Interestingly, we find that the hub oscillation period *T* determines the length of the attractor cycle *L* and that networks learn a target function best when the input period *T* is equal to the length *L*_*c*_ of the target function (*T*≈*L*_*C*_, see **Fig N in S1 Text**).

Since networks can exhibit different attractor cycles in response to different input hub frequencies, we ask whether these networks are also capable of time-division multiplexing, i.e. learning different target functions when the network hub is exposed to different input periods. Concretely, time-division multiplexing means that we challenge networks to map a specific input oscillation period of the hub to learning a corresponding target. In this setting, the network starts from completely random initial condition states during each generation to probe whether hub oscillations alone are sufficient for time division multiplexing. Intuitively, the problem we want to address now is the following. Imagine that we have *M* different target functions $f_{1}=\{{\hat{\sigma }}_{1}(t_{1}),\cdots ,{\hat{\sigma }}_{1}(t_{L_{1}})\},\;f_{2}=\{{\hat{\sigma }}_{2}(t_{1}),\cdots ,{\hat{\sigma }}_{2}(t_{L_{2}})\},\cdots ,f_{M}=\{{\hat{\sigma }}_{M}(t_{1}),\cdots ,{\hat{\sigma }}_{M}(t_{L_{M}})\}$, and we force the network, once at a time, with *M* different periodic signals with periods *T*_1_, *T*_2_,⋯,*T*_*M*_. Each external signal is applied starting the dynamics from a random initial condition. What changes from one realization to another are the initial condition and the external signal applied to the hub. Is it possible to make the network learn the *M* different target behaviors by applying *M* different external oscillating signals? If so, what are the conditions the periods of the forcing signals must fulfill to make the network adapt to *M* different responses?

Importantly, because the specific periods of the oscillating hub determine the length of the new resonant attractors, we restrict ourselves to setting the length of each target function to be the same as each different hub oscillation period. We generate a set of *M* target functions of length *L*_1_, *L*_2_,⋯*L*_*M*_ and we chose a set of oscillatory input functions with pre-defined periods *T*_1_, *T*_2_,⋯*T*_*M*_ that will correspond to the length of each target function, namely, with *L*_*i*_ = *T*_*i*_. Each oscillatory function is applied to the hub node of the network, and we allow the network to converge upon an attractor cycle; the network is reset with different random initial conditions and the next oscillation function is applied. Additionally, to enforce learning multiple functions simultaneously, we take the mean squared error across the different input periods-target functions pairings to be the fitness (**Fig 3B)**. The input hub and target node for each network in the population are the same throughout the learning (adaptation) process.

We find that these networks can learn several distinct target functions by simply varying the period of the oscillating hub during the learning cycles to minimize the Hamming distance *h* between all target-actual behavior matches. We hereafter refer to this network-based learning procedure as *resonant learning*. We see that the network learns shorter timescale targets first and the longer timescale targets afterwards (**Fig 3C**). This hierarchy disappears when the number *M* of functions with different timescales increases, the network then struggles to learn the longer time scale functions as well as those with shorter timescales (**Fig 3C).** Furthermore, if we challenge the network to learn different targets to match different input sequences of the hub (all of period *T* = 10), we find that the fitness of the function is no longer able to converge for *M*>7, indicating that 7 functions may be an upperbound for the capacity of networks used in this analysis (**Fig O in S1 Text**). The important point is not, however, the specific value *M* = 7 of the upper bound, but the fact that such an upper bound exists.

We now compare the learning efficacy under two other alternative evolutionary schemes: forcing the hub node to stay constant on the one hand and leaving it free to follow the rule in Eq (1) on the other hand. In both cases we allow the hub node’s outgoing weights to be mutated during learning (as with the oscillatory input signal case). Since now the hub node will either remain constant or be free to follow the updating rule in Eq (1), we cannot differentiate the behavior of the hub node by means of input signals with different periods. Therefore, instead of using *M* input oscillations with different frequencies, now the network multiplexing learning is implemented by starting the dynamics from *M* different predefined initial conditions (**Fig 3D)**. Surprisingly, we find that with this procedure, networks were unable to learn any of the target functions with the same efficiency as with resonant learning in the presence of an oscillatory hub (**Figs 3E and P in S1 Text)**. Even for the most straightforward task of learning three target functions, the resonant learning scheme converges at least one order of magnitude faster than learning these three target functions with a non-oscillating hub. We attribute poor learning in presence of a fixed hub state to the network’s inability to learn longer target cycles without an oscillating, periodic input that forces the network to create attractor cycles of the same length than that of the target functions.

**Fig 4:**
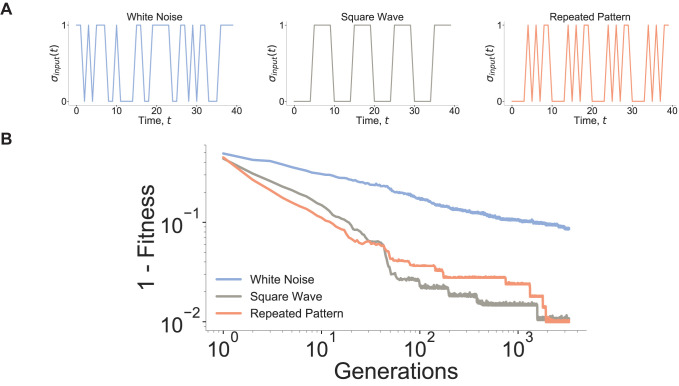
Learning is solely dependent on the timescale of oscillations. **(A) Different periodic hub input time series.** Three different input types are defined: square wave inputs which we have used previously, a white noise pattern with a set sequence and no characteristic timescale, and a repeated pattern input with a fixed period. (**B) Input period oscillation is more important than input pattern.** Multiplexed learning of three different target functions of length 8, 10, 12 for networks *N* = 500, *γ* = 1.9 on the three types of input sequences are shown. Repeated pattern inputs and square wave inputs yield faster learning than white noise inputs, showing that the timescale associated hub oscillations matter more than the specific repeated pattern.

Next, instead of using periodic square wave functions to force the state of the hub, we ask whether other periodic but irregular patterns could give an advantage in learning. (For instance, we take as the input signal a random sequence of length *T* and repeat it to make it periodic with period *T*.) First, we test a simple learning case in which the networks must learn three different target functions of lengths {*L*_1_, *L*_2_, *L*_3_} = {8,10,12}. As shown above, networks can easily learn these different target functions when we use matching square wave input periods {*T*_1_, *T*_2_, *T*_3_} = {8,10,12}. By contrast, we show that when we use periodic but random patterns of 0 and 1s of length {*T*_1_, *T*_2_, *T*_3_} = {8,10,12}, instead of regular square waves, the network can still learn efficiently, indicating that the timescale, not the specific repeated pattern, associated with the oscillations of the input may be the key feature for resonant learning (**Fig 4A and 4B)**. An alternative explanation is that, throughout the evolutionary process, the network can incorporate any specific input hub state sequence in order to learn (adapt) any target function, rather than recognizing the periodicity of the signal introduced onto the hub node. To rule out this hypothesis, during the learning process involving three different target functions of lengths {*L*_1_, *L*_2_, *L*_3_} = {8,10,12}, we used as input signals forcing the hub three distinct time series whose sequences display no specific timescale and resemble white-noise (with no periodicity whatsoever). Under these conditions, we find that learning performance is degraded when we use white noise as input functions in the absence of a typical timescale (**Fig 4A and 4B)**.

Since the specific pattern of the oscillatory input signal at the hub is not central for the network to learn the target function efficiently, we ask if the network is capable of learning multiple target functions of the same length as a function of different patterned inputs in one evolutionary run. Indeed, we find that the networks can learn multiple target functions to match inputs all of length *10* **(Fig O in S1 Text)**. By contrast, when the targets vary in length, the network fully learns the shorter length targets before learning target functions with longer periods (**Fig 3B–3D**). Altogether, these results led us to propose that the well-defined timescale associated with the input period is the key feature that governs resonant learning.

## Discussion

Cells and other complex dynamical systems are often subjected to oscillating external signals. The evolution and adaptation of such systems in the presence of periodic stimuli require internal reconfiguration of their dynamic networks to cope with the environmental challenges associated with these periodic signals. For instance, calcium oscillations in the extracellular medium trigger essential functions of the sperm cell to fertilize the egg [44]. Immune T cells can selectively filter out the frequency of external periodic signals to trigger different inflammatory responses [17,45]. An important and still outstanding problem is to determine the evolutionary mechanisms through which cells can develop new functions in the presence of oscillatory signals. Cells can evolve and adapt to almost any environment, particularly an oscillating one. But how a cell (or any other complex dynamical system) can take advantage from an oscillating environment to evolve and develop new functions is still unclear.

In this work, we use Boolean threshold networks as well-established prototype models for gene regulation networks and signaling pathways. We demonstrate that by forcing the network hub to oscillate we can induce a wide variety of novel attractor cycles, which can be interpreted as new phenotypes or dynamical states of the cell. Furthermore, not only can such input oscillations select and force a network to perform a novel behavior, but they also confer a distinct evolutionary learning advantage, which is that the network can more easily and efficiently learn new predefined target behaviors when the oscillating external signal is present than when it is absent.

Our results provide a plausible explanation for recent biological observations [6,7] suggesting that master regulatory nodes may be uniquely poised to coordinate entire network behavior simply through pulsatile inputs in a frequency domain, rather than time domain. Our results also support experimental observations showing that cells can develop new phenotypes by filtering out the frequency of input signals instead of the amplitude [17]. To achieve this behavior, the network does not have to change its entire attractor (or epigenetic) landscape. New attractors emerge because of the external periodic perturbation while preserving the ground-state ones. This is a typical behavior of networks operating at the critical point. In fact, this is also the reason why we choose to work with networks operating at the critical regime, as it has been demonstrated that critical networks can simultaneously combine robustness (preservation of old phenotypes) and adaptation (the emergence of new phenotypes) in the face of external perturbations [36,46].

More generally, our observed *resonant learning* phenomenon opens the door to new strategies for how to control complex network behaviors with access to only a few nodes such as hubs. Surprisingly, *resonant learning* outperforms similar learning procedures in the absence of oscillating inputs for comparable tasks by orders of magnitude. Previous studies demonstrated that topological separation into distinct domains (or motifs) of the network architecture was necessary for learning multiple tasks [47,48]. By contrast, we discover here an alternative and complementary procedure by which a network can learn multiple tasks by relying upon the dynamical properties of the network as opposed to only static network structures. This finding may have important implications for the understanding of the evolutionary mechanisms that are into play in changing environments. Our results show that learning is optimal (resonant) when the timescale of the environmental periodic signal coincides with that of the new target attractor that needs to be learned. Consequently, a single network can learn a range of new attractors that have different timescales. As demonstrated earlier, the network’s hub needs to be excited by oscillatory signals with respectively same timescales as those that define the new learned attractors. Naturally, the number of different attractors that can be learned is limited by the longest timescale in response to the hub oscillatory signals, which we reported as the relaxation time.

Altogether our results show that *resonant learning* is a potentially useful property, however, in this work we have not directly applied it to known and resurging machine learning frameworks. Rather, our goal is not to generate the most efficient algorithm to make the network learn any particular task, but to characterize the role that oscillatory environments may play in the evolution of organisms and complex dynamical systems. From a biological perspective, our results suggest that oscillating environments can potentiate the ability of regulatory and signaling networks to adapt to new challenges by evolving the appropriate responses. Importantly, criticality plays a crucial role in this learning process. Networks operating in the ordered regime are too ‘stiff’ and do not exhibit enough dynamical richness to develop new functions. Networks in the chaotic regime are too sensitive to external stimuli and constantly change their behavior, making them unable to preserve the acquired functionality. By contrast, our results show that Boolean networks operating near the critical regime can learn faster and more efficiently new tasks based on the frequency of the input signal than in the absence of the oscillatory signal. Overall, our study sheds light on a novel property of large dynamical systems and how evolution may have exploited it to select and learn new dynamical behaviors.

## Supporting information

S1 Text**Fig A. Dynamical updating rule**. We define the network to have a simple thresholded activation function to determine the state of the node *σ*_*i*_(*t*+1) as defined in Eq (1) of the main text. For each connection *σ*_*i*_→*σ*_*j*_, the connection weight *w*_*ij*_ is randomly chosen with uniform probability in the interval [–1,1] and kept fixed through the temporal dynamics of the network. To determine the value *σ*_*i*_(*t*+1) for each node in the network at time *t*+1, we have to know the states at time *t*, *σ*_*j*_(*t*), and the weights of all nodes that have an out-going edge to *σ*_*i*_. While simple, this updating rule has several advantages over the more classical Kauffman networks, which require a truth table of memory *O*(*N*2^*K*^) to define the updating rules for each of the *N* genes in the network (each with average connectivity *K*), allowing us to simulate larger random networks efficiently. **Fig B.** Threshold networks exhibit a continuous phase transition from ordered to chaotic states even with hub node oscillations. Because the threshold activation function makes analytical approximation intractable, we instead evaluate this phase diagram empirically through simulations using the Hamming distance as the order parameter: $\underset{t\to \infty }{\lim}h(t)=\underset{t\to \infty }{\lim}\frac{1}{n}\sum_{i=1}^{n}\left|\sigma_{i}(t)-{\tilde{\sigma }}_{i}(t)\right|$. We perturb a fraction, *d = 0*.*05* of the states in the network and calculate the difference in trajectories between the perturbed initial condition, ${\tilde{\sigma }}_{}(t)$, and the original condition, ***σ***(***t***). For each *γ*, we average ⟨*h*(*t*)⟩ over five different initial conditions and twenty different networks. Networks are constructed with scale free topologies and N = 500 nodes. Oscillations are applied to the most connected hub node with different square wave periods. As confirmed by Zañudo *et al*. [32], these Boolean threshold networks are such that ⟨*h*(*t*)⟩ approaches 0.2 in the chaotic regime, rather than 0.5, due to many nodes in the network freezing in either the 0 or 1 state. **Fig C.** The corresponding time series for the frequency domains shown in Fig 1B. Here, we color the output states when they are synchronized with the input state. **Fig D.** The power spectra of input and network nodes, as shown in Fig 1C, with an input period of T = 8. The top plot shows the power spectra of the input node with the overlaid square oscillation in the time domain. The bottom trace shows the power spectra of the time series for all nodes in the same network. To calculate the power spectra for a given node’s time series in the Boolean networks, we sample only the parts of their time series in which the node is in a known attractor. We extract the states of a given node, *σ*_*i*_(*t*_*start*_), *σ*_*i*_(*t*_*start*_+1),…,*σ*_*i*_(*t*_*end*_), We use the built-in *Periodogram* function in the Scipy signal library of Python to estimate the power spectra of these signals for comparison. We plot only the dominant frequency. **Fig E. Overlap between new attractors states.** When creating new attractor cycles with oscillations, we considered how different the newly created attractor cycle states were with respect to each other. It would be uninteresting if oscillating the input node at two separate frequencies resulted in two attractors incorporating the same network states. We tested this by generating 20 different networks for each gamma value and two oscillation periods, T = 4 and T = 6. We probed the attractor landscape to identify up to 20 attractors and, for each period, found the most similar attractor cycle in the landscape corresponding to the other period. We find that for *γ*<2.3, the average overlap between attractors created by a slight difference in input oscillation is low, indicating that we generate novel attractor cycles for different input signals. **Fig F.** In addition to cataloging similarity between the new attractor states for different periods of input oscillation, we compare the new attractor states to attractor states when the input, σ_input_ is blocked. Let *A*_*T*_ be the set of all network configurations **σ**_**i**_ such that **σ**_**i**_ is part of an attractor cycle when σ_input_ oscillates with period *T*, and let *B* represent the set of all network configurations **σ**_**j**_ such that **σ**_**j**_ is part of an attractor cycle when σ_input_ is blocked at OFF or ON. We calculate $1-\frac{\left|A_{T}\cap B\right|}{\left|A_{T}\right|}$averaged over 10 different networks and 1000 initial conditions for each value of *γ* to identify the attractor landscape. As the input period increases beyond the relaxation timescale of the network, there is always some overlap with the control landscape. **Fig G.** New attractor cycles are robust. We quantify in 1,000 initial conditions the fraction of unique attractor cycles are found, which we define as the attractor landscape density. Again, we average over 10 different networks for each *γ*. We find that the new attractor cycles constructed from input oscillations in low-*γ* networks are indeed robust, as the attractor landscape is sparse (i.e., multiple initial conditions converge on the same attractor). Note, for clarity of presentation, we add 0.02 to the bars for gamma = 1.7 in order to see that these values are non-null. There are indeed some attractors discovered. **Fig H.** Error in the learning process of the network throughout generations (see also Fig K **in S1 Text**). The learning consists in evolving the network so that the temporal signal of one node chosen randomly, (the output node), matches a predefined function (the target function). The error (*E* = 1−*Fitness*) is the difference between the actual temporal signal of the output node and the target function (see Fig A **in S1 Text**). It can be observed that throughout generations the error decreases, which means that the networks learn to reproduce the target function with increasing precision. We report here the results for networks of different size. In each case the network was evolved to pair three different input signals of period *T = {6*,*8*,*10}* to match targets target functions with the same periods. This result demonstrates that networks with *N* = 500 and *N* = 1000 learn essentially at the same rate. **Fig I. Testing multiple gamma values.** Error in the learning process throughout generations for networks with different values of the scale-free exponent *γ*. The learning process is the same as described in the main text and in Figs H and K **in S1 Text**. It can be observed that networks with lower values of *γ* are able to learn significantly faster, particularly for *γ*<1.9, than networks with larger vaues of *γ*, justifying our choice of *γ* = 1.9 in most of our simulations. **Fig J. S10 Fig: Average size of attractor cycles**. We catalogue in the probed attractor landscapes the average size of each attractor found. In chaotic networks, the size of the attractors is far larger than in ordered networks, becoming unreasonable to simulate for γ < 1.7. Additionally, we show the average attractor size when the hub node is allowed to update naturally as a function of other nodes in the network and when the hub input node is set to a constant, blocked value of ON (OFF). **Fig K. Schematic illustration of the network learning process.** A periodic input signal with period *T* is introduced into the hub of the network (red node). A randomly selected node (other than the hube) is chosen to be the output node (green node). During the temporal dynamics of the network (Eq (1) of the main text) the output node will generate an output signal *σ*_*out*_(*t*) that depends on the specific details of the networks (connections and weights), and on the form of the input function. The output signal *σ*_*out*_(*t*) is then compared with a predefined target function *f*(*t*) which can or cannot be periodic (in the illustration, the target function is periodic, but this is not necessaryly the case). Both the input and target functions are computed for a period *L* of time. The error *E* between *σ*_*out*_(*t*) and *f*(*t*) is computed as the normalized Hamming distance between these two functions averaged over the time interval *L*. **Fig L. Schematic illustration of the evolutionary algorithm.** Beginning with a population of *N*_*pop*_ = 50 networks, we generate three mutants for each network. Mutations consist in randomly rewiring the network connections and/or changing the connections weights. The mutation rate is *μ* = 0.02 for each node in the network. Then, we calculate the fitness function of each of our networks in the population and select only the best *N*_*pop*_ = 50 networks, which are the ones that pass to the next generation. This reduces the population to its original size. One full process of mutations and selection represents one generation. **Fig M. Limitations on learning.** We demonstrate that networks with N = 500 and γ = 1.9 struggle to learn specific attractor cycle behaviors for long time scale input periods. Specifically, we assayed learning for input periods, *T*, and target cycle length, *L*, *T = L = {6*,*30*,*40}*. As can be seen, the network is only able to completely learn the short timescale of *T = L = 6*, demonstrating the limitation of resonant learning for targets longer than the relaxation time (t_relax_ = 20) of the network. We clarify that t_relax_ = 20 is half a single period, so this corresponds to T = 40. **Fig N.** Learning target functions with lengths that differ from the period of the input function. Here, we find that the network learns best when the input period and target are of equal duration. Interestingly, even having a divisor of the target cycle length does not yield the same benefit in learning, as is the case for *T* = 2, *L* = 10). Learning performance worsens when the target is longer than the input period and is not a multiple of it (*T* = 8, *L* = 10). We have a slight improvement in learning performance when the target period is shorter than the input length (*T* = 10, *L* = 8). **Fig O. Learning several patterns.** We force the network to learn repeated input functions of size *T* = 10 to output functions of the same length *L* = 10. Because the inputs are arbitrary repeated patterns, our fitness function can allow the network to learn different repeated patterns of the same length without compromising other repeated pattern inputs. This result shows that the network’s ability to learn an arbitrary number of repeated patterns considerably degrades when the number of patterns is larger than 7. **Fig P. Learning multiple target functions of varied length when the input node is under the same dynamical rule as the rest of the network**. We repeat the same procedure as for Fig 3D but allow the input node’s dynamics to follow the same updating rule as the rest of the network (Eq (1) of the main text), giving the network more control over its behavior. We seek to level the playing field between the oscillating input case, where the network has a signal from the hub (Fig 3C and 3D of the main text). While learning improves, the network cannot learn long targets, yielding poor performance when learning multiple attractor functions.(DOCX)
