# Supplementary material for: Resonant learning in scale-free networks
Source: PLoS Comput Biol. 2023 Feb 21;19(2):e1010894. doi: 10.1371/journal.pcbi.1010894 (PMC9983844; doi:10.1371/journal.pcbi.1010894)
Supplement: S1 Text — Fig A. Dynamical updating rule. We define the network to have a simple thresholded activation function to determine the state of the node σi(t+1) as defined in Eq (1) of the main text. For each connection σi→σj, the connection weight wij is randomly chosen with uniform probability in the interval [–1,1] and kept fixed through the temporal dynamics of the network. To determine the value σi(t+1) for each node in the network at time t+1, we have to know the states at time t, σj(t), and the weights of all nodes that have an out-going edge to σi. While simple, this updating rule has several advantages over the more classical Kauffman networks, which require a truth table of memory O(N2K) to define the updating rules for each of the N genes in the network (each with average connectivity K), allowing us to simulate larger random networks efficiently. Fig B. Threshold networks exhibit a continuous phase transition from ordered to chaotic states even with hub node oscillations. Because the threshold activation function makes analytical approximation intractable, we instead evaluate this phase diagram empirically through simulations using the Hamming distance as the order parameter: limt→∞h(t)=limt→∞1n∑i=1n|σi(t)−σ~i(t)|. We perturb a fraction, d = 0.05 of the states in the network and calculate the difference in trajectories between the perturbed initial condition, σ~(t), and the original condition, σ(t). For each γ, we average ⟨h(t)⟩ over five different initial conditions and twenty different networks. Networks are constructed with scale free topologies and N = 500 nodes. Oscillations are applied to the most connected hub node with different square wave periods. As confirmed by Zañudo et al. [32], these Boolean threshold networks are such that ⟨h(t)⟩ approaches 0.2 in the chaotic regime, rather than 0.5, due to many nodes in the network freezing in either the 0 or 1 state. Fig C. The corresponding time series for the frequency domains shown in Fig 1B. Here, we color the out [file pcbi.1010894.s001.docx]

**Supplementary Information**

**Resonant Learning in Scale-free Networks**

Samuel Goldman^a^, Maximino Aldana^b,^*and Philippe Cluzel*

Department of Molecular and Cellular Biology, Harvard John A. Paulson School of Engineering and Applied Sciences, Harvard University, Cambridge, MA 02138, USA.

^a^Current address: MIT Computational and Systems Biology.

^b^ Instituto de Ciencias Fisicas, Universidad Nacional Autonoma de Mexico, Cuernavaca, Morelos 62210, Mexico, Centro de Ciencias de la Complejidad, Universidad Nacional Autónoma de México. Coyoacán 04510. Mexico City, Mexico.

*Corresponding authors

**Fig A in S1 Text**

**Fig B in S1 Text**

**Fig C in S1 Text**


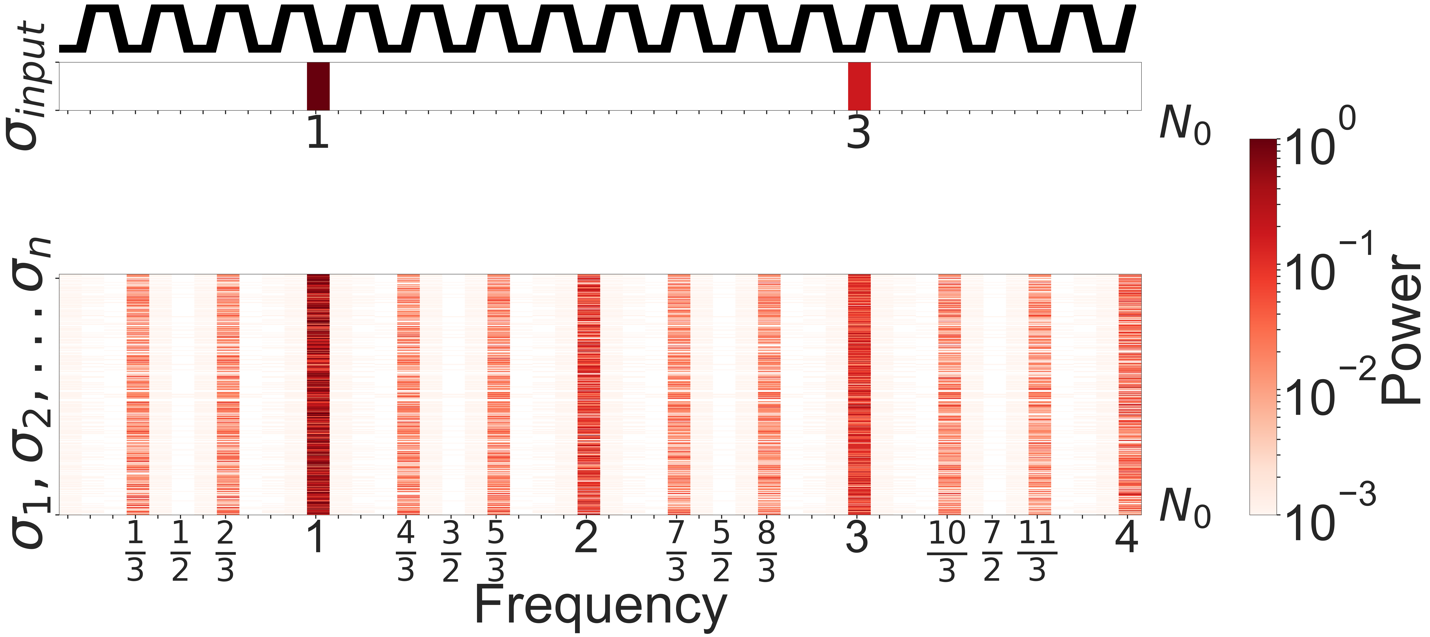


**Fig D in S1 Text**

**Fig E in S1 Text**

**Fig F in S1 Text**

**Fig G in S1 Text**

**Fig H in S1 Text**

**Fig I in S1 Text**

**Fig J in S1 Text**


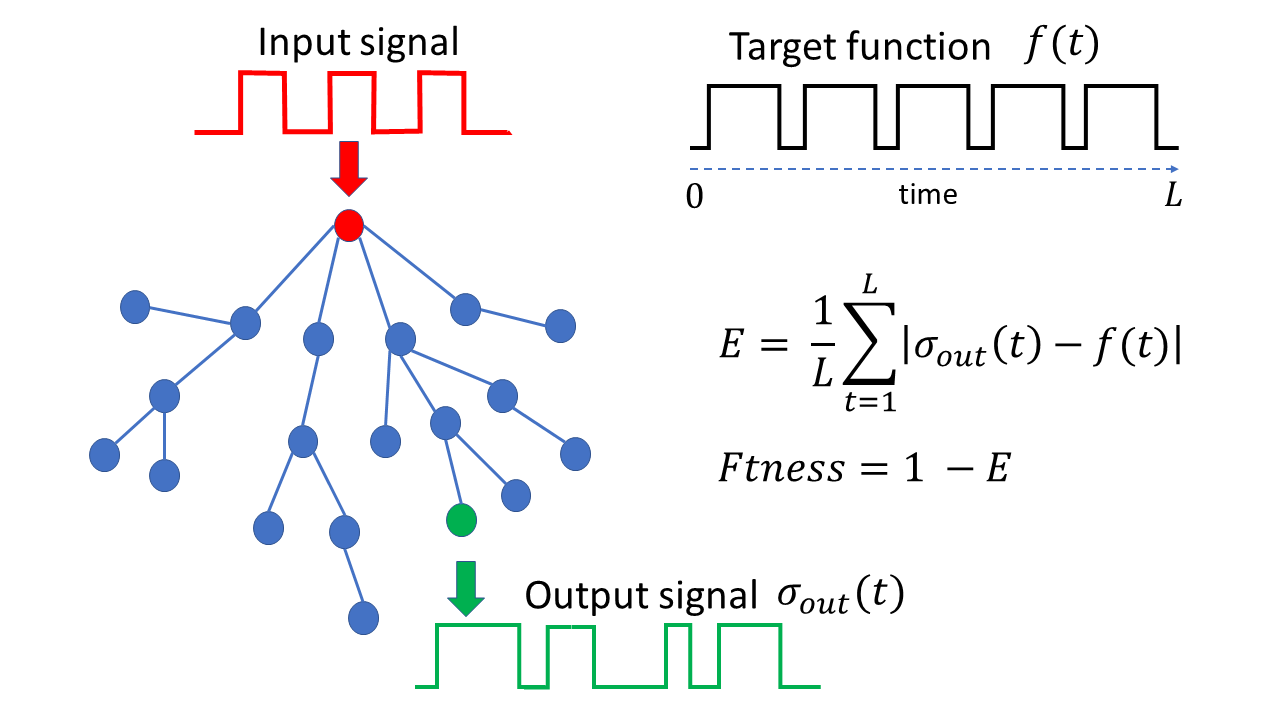


**Fig K in S1 Text**

**Fig L in S1 Text**

**Fig M in S1 Text**

**Fig N in S1 Text**

**Fig O in S1 Text**

**Fig P in S1 Text**

**Defining an attractor.** Since both the network connections $\sigma_{i}\to\sigma_{i}$ and the connection weights $w_{ij}$ are determined when the network is constructed and do not change in time, the network dynamics given by Eq. (1) of the main text are deterministic. Additionally, a network with $N$ nodes has a finite number of $2^{N}$ states. The configuration space is finite. Therefore, after some transient time, the network inevitably will fall in a previously visited state. The dynamics will repeat from that point on, making the network fall into a periodic pattern of activity. In the first part of our results, when discussing the attractors of the network, we include the input node as part of this attractor definition. Therefore, if the input node oscillates with an input period $T=4$, the attractor must, by definition, be a multiple of 4. To allow for more general results, when forcing these networks to learn different attractor states, we remove this restriction from the definition of the attractor. We generally define the attractor as the expression pattern of all nodes, excluding the input node (the hub) [1-5].

**References:**

1. Catanzaro, Michele, Marián Boguná, and Romualdo Pastor-Satorras. "Generation of uncorrelated random scale-free networks." *Physical review e* 71.2 (2005): 027103.
2. Derrida, Bernard, and Yves Pomeau. "Random networks of automata: a simple annealed approximation." EPL (Europhysics Letters) 1.2 (1986): 45.
3. Oikonomou, Panos, and Philippe Cluzel. "Effects of topology on network evolution." *Nature Physics* 2.8 (2006): 532.
4. Park, Jin, et al. "Molecular time sharing through dynamic pulsing in single cells." *Cell systems* 6.2 (2018): 216-229.
5. Zanudo, Jorge GT, Maximino Aldana, and Gustavo Martínez-Mekler. "Boolean threshold networks: Virtues and limitations for biological modeling." *Information Processing and Biological Systems*. Springer, Berlin, Heidelberg, 2011. 113-151.
